# Risk of Breast Cancer in Women with Mastitis: A Retrospective Population-Based Cohort Study

**DOI:** 10.3390/medicina56080372

**Published:** 2020-07-24

**Authors:** Ying-Cheng Chen, Chi-Ho Chan, Yu-Bing Lim, Shun-Fa Yang, Liang-Tsai Yeh, Yu-Hsun Wang, Ming-Chih Chou, Chao-Bin Yeh

**Affiliations:** 1 Institute of Medicine, Chung Shan Medical University, Taichung 402, Taiwan; chenyc66@gmail.com (Y.-C.C.); coolim_28@hotmail.com (Y.-B.L.); ysf@csmu.edu.tw (S.-F.Y.); 68990@cch.org.tw (L.-T.Y.); 2 Department of Surgery, Changhua Christian Hospital, Changhua 500, Taiwan; 3 Department of Microbiology and Immunology, Chung Shan Medical University, Taichung 402, Taiwan; chiho@csmu.edu.tw; 4 Department of Medical Research, Chung Shan Medical University Hospital, Taichung 402, Taiwan; cshe731@csh.org.tw; 5 Department of Surgery, Chung Shan Medical University Hospital, Taichung 402, Taiwan; 6 Department of Anesthesiology, Changhua Christian Hospital, Changhua 500, Taiwan; 7 Department of Emergency Medicine, School of Medicine, Chung Shan Medical University, Taichung 402, Taiwan; 8 Department of Emergency Medicine, Chung Shan Medical University Hospital, Taichung 402, Taiwan

**Keywords:** breast cancer, risk factors, mastitis

## Abstract

*Background and objectives*: Breast cancer is a common cancer in women and has been the fourth leading cause of death in Taiwanese women. Risk factors for breast cancer include family history of breast cancer, genetic factors, and not breastfeeding. Several studies have reported an association between repeated inflammation at a young age, especially among lactating women, and cancer; however, the number of studies about the association of mastitis and breast cancer in nonlactating women is still limited. Therefore, the aim of this study was to determine the relationship between mastitis in women aged ≥40 years and breast cancer. *Materials and Methods:* This was a retrospective cohort study design. The data source was the Longitudinal Health Insurance Database 2010 (LHID 2010), comprising data collected by Taiwan’s National Health Insurance program. Cases of newly diagnosed mastitis in women aged ≥40 years (ICD-9-CM code = 611.0) were selected from the years 2010 to 2012. Women not diagnosed with mastitis were selected as the control group, and their data for the years 2009 to 2013 were obtained through the database. In addition, the non-mastitis group was matched 1:10 by age. *Results*: A total of 8634 participants were selected from the LHID 2010, which included 734 cases with mastitis and 7900 cases without mastitis. After adjustment for age, hypertension, hyperlipidemia, diabetes, hypothyroidism, and autoimmune diseases, the Cox proportional hazard model showed that patients with mastitis had a higher risk of breast cancer (aHR = 3.71, 95% CI = 1.9–7.02) compared with the non-mastitis group. The Kaplan–Meier curve also showed that women with mastitis had a higher risk of developing breast cancer. *Conclusions*: This study confirmed that women with mastitis have a higher risk of developing breast cancer. Therefore, women aged ≥40 years could reduce breast cancer risk by taking precautions to prevent mammary gland infection and mastitis.

## 1. Introduction

Breast cancer is one of the most common types of cancer in women [1]. If surgery is performed during the first stage of breast cancer, the five-year survival rate can be as high as 89.5%. Despite this, breast cancer remains the fourth leading cause of death in Taiwanese women [2,3]. For early prevention of breast cancer in women, it is critical to identify the cancer-related risk factors to encourage high-risk groups to take appropriate precautions.

Mastitis is the inflammation of the mammary gland, and it can have multiple etiologies. For example, bacterial infection is associated with mastitis, with breastfeeding being a possible portal of entry for pathogens in lactational mastitis. According to estimates by the World Health Organization, 10–30% of lactational mastitis occurs in postpartum women [4]. The predominant species of bacteria involved in mastitis includes *Staphylococcus aureus*, *S. epidermidis* and *Corynebacterium* spp. [5]. In addition, granulomatous mastitis might be associated with different *Corynebacterium* species [6].

Many studies have indicated that inflammation increases the risk of developing different types of cancers. According to one population-based study, patients with inflammatory bowel diseases have increased risk of small bowel cancer. Due to the complexity of immunological stimulation, chronic inflammation is an important process in the oncogenesis of breast cancer [7]. Some studies have shown that the use of aspirin and other nonsteroidal anti-inflammatory drugs (NSAIDs) lower the risk of colorectal cancer, breast cancer, and skin cancer [8,9,10,11]. Although the regular use of aspirin and other NSAIDs is reported to reduce the risk of breast cancer, one study found that low-dose aspirin was not associated with improved breast cancer prognosis [12].

Lambe et al. showed that women who had a record of mastitis would be mildly more at risk of breast cancer [13]. Moreover, Chang et al. reached a similar conclusion in their study with a population of adult women [14]. Furthermore, after conducting a systemic review, Nolan et al. recently presented the hypothesis that mastitis might be a risk factor for breast cancer [15]. Therefore, the aim of this study was to investigate whether patients aged ≥40 with a history of mastitis have an increased risk of developing breast cancer. We hypothesize that elderly women with mastitis have significant differences in breast cancer risk.

## 2. Material and Methods

### 2.1. Data Sources

The National Health Insurance Research Database (NHIRD) is maintained by the Taiwan National Health Research Institutes. It contains data on more than 99% of the 23 million residents of Taiwan enrolled in National Health Insurance. The Longitudinal Health Insurance Database (LHID) contains one million beneficiaries randomly sampled from the database. There are no differences in the LHID sample with respect to the distribution of age or gender in the entire population. The database contains all outpatient and inpatient medical claims, including drug medications, medical operations, procedures, and fees. This study was approved by the Ethical Review Board of Chung Shan Medical University Hospital (CS 18096).

### 2.2. Study Group and Outcome

This was a retrospective cohort study. We recruited female patients from the LHID. The study population was newly diagnosed with nonlactating mastitis (ICD-9-CM code = 611.0) from 2010 to 2012 and aged ≥40 years. The National Health Insurance had a review mechanism to confirm the reliability of disease diagnosis. The index date was the first diagnosis date of mastitis. We excluded diagnoses of breast cancer (ICD-9-CM code = 174) before the index in order to confirm new-onset disease. The non-mastitis patients were defined as not having had a diagnosis of mastitis (ICD-9-CM codes = 611.0, 675) from 2009 to 2013. A 1:10 matching by age was used to provide an index date for the non-mastitis patients that had the same starting point. Furthermore, we excluded women who were pregnant 1 year before the index date up to the end date. The outcome variable was defined as a diagnosis of breast cancer (ICD-9-CM code = 174). Both groups were followed up until the onset of breast cancer, until 31 December 2013, or until withdrawal from the social insurance program, whichever occurred first. In addition, to avoid breast cancer that occurred in a short period of time due to unmeasured factors, the follow-up duration was defined as being a minimum of 6 months.

### 2.3. Covariates

The baseline characteristics were age, hypertension (ICD-9-CM codes = 401–405), hyperlipidemia (ICD-9-CM codes = 272.0–272.4), diabetes (ICD-9-CM code = 250), hyperthyroidism (ICD-9-CM code = 242), hypothyroidism (ICD-9-CM code = 243, 244), and autoimmune diseases (ICD-9-CM codes = 710.0, 710.2, 720.0, 714.0). The comorbidities were defined within 1 year prior to the index date.

### 2.4. Study Covariates

To compare the characteristics of mastitis and non-mastitis patients, the Student’s *t*-test and chi-squared test were calculated, as appropriate. Kaplan–Meier analysis was used to calculate the cumulative incidence of breast cancer among the two groups. The log-rank test was used to test the significance. To determine the independent risk of mastitis, the multivariate Cox proportional hazard model was used to estimate the hazard ratios. The statistical software version used was SPSS 18.0 (SPSS Inc., Chicago, IL, USA).

## 3. Results

**Figure 1:**
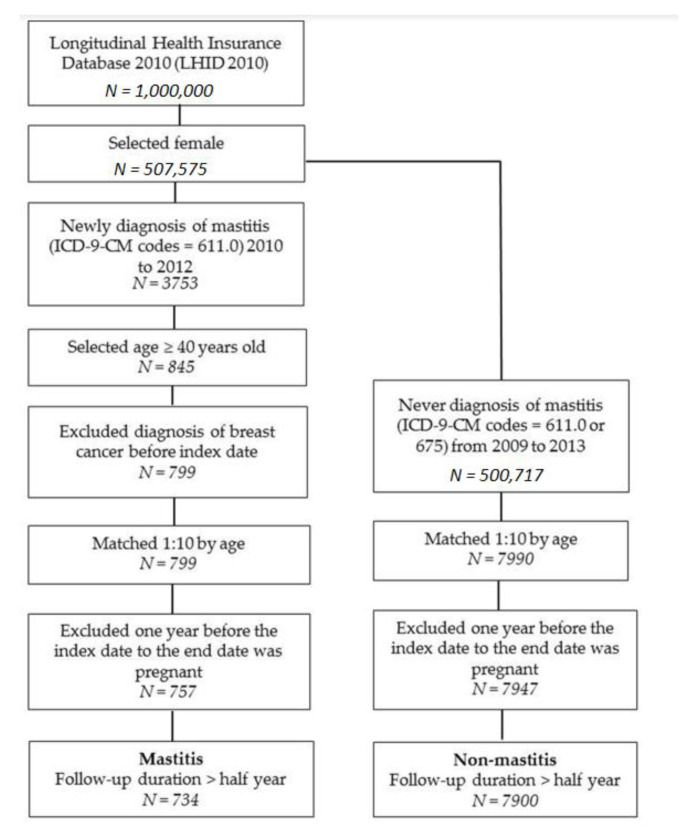
Flow chart for patient selection.

There were 734 mastitis patients and 7900 non-mastitis patients enrolled in the study (Figure 1). The distribution of age and incidences of hypertension, hyperlipidemia, diabetes, hyperthyroidism, hypothyroidism, and autoimmune diseases are presented in Table 1. The mean ages (and standard deviations) of mastitis and non-mastitis patients were 51.1 (SD: 8.7) and 50.7 (SD: 8.8) years old, respectively. The mastitis group had higher proportion of hypertension (20.6%) and hyperlipidemia (16.1%). Besides, the mean ages and incidences of comorbidities were similar between the two groups.

**Figure 2:**
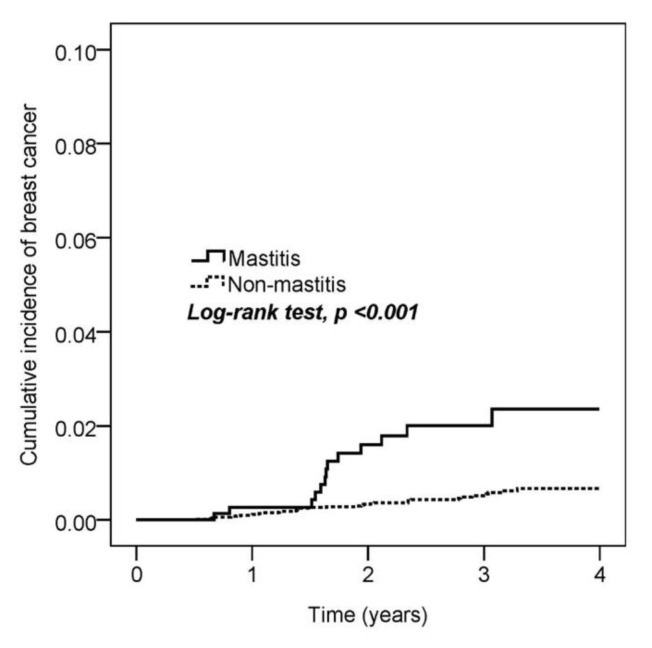
Kaplan–Meier curves of the cumulative proportion of breast cancer.

Next, we estimated the cumulative incidence of breast cancer among mastitis and non-mastitis patients. The Kaplan–Meier curve indicated that mastitis patients had a higher risk of breast cancer (log-rank test, *p* < 0.001; Figure 2). The incidence densities in the mastitis and non-mastitis groups were 6.8 and 1.7 per 1000 person-years, respectively. In the Cox proportional hazard model, mastitis patients (HR = 3.71, 95% CI = 1.96–7.02) and comorbidity of hyperlipidemia (HR = 2.53, 95% CI = 1.15–5.57) had a significantly higher risk of breast cancer (Table 2).

We also conducted subgroup analyses between mastitis and non-mastitis patients. Mastitis patients aged 20–50 had a significantly increased risk of breast cancer (HR = 2.66; 95% CI = 1.00–7.05). Greater risks of breast cancer events were observed in mastitis patients aged ≥50 years (HR = 5.46; 95% CI = 2.31–12.87). Within the mastitis group, the subgroup of mastitis patients with comorbidities was observed to have a higher risk of breast cancer. Due to the number of breast cancer cases, some results were statistically unavailable (Table 3).

## 4. Discussion

Women newly diagnosed with mastitis and aged ≥40 were recruited for observation of breast cancer risk. Our study indicates that, in Taiwan, women who have had mastitis have a higher risk of developing breast cancer, particularly women aged ≥50 years. In addition, women who had comorbidity, such as hyperlipidemia, had an increased risk of breast cancer. Our findings are consistent with previous studies [13,14]. In their 2009 cohort study, Lambe et al. identified from their database 8411 women who had a diagnosis of mastitis. Among these women, 106 had a subsequent diagnosis of breast cancer. When women with breast cancer were compared with those who had no recorded mastitis, the incidence rate of breast cancer was higher in women with mastitis [13]. Another study conducted by Chang et al. identified that women with nonlactational mastitis faced a higher risk of breast cancer [14], which is similar to our results. In addition, our study focused on women aged ≥40. Therefore, this strategy could reduce the pregnancy effect, which might interrupt the risk of breast cancer.

Several risk factors for breast cancer have been identified. Literature reviews have aggregated all known risk factors for breast cancer; apart from genetic factors such as BRCA 1/2 and other mutated genes, aging (i.e., aged >40 years), family history (i.e., breast cancer in mother/sister), lifestyle (i.e., alcohol consumption and smoking), estrogen administration (i.e., hormone therapy), and reproductive factors (i.e., early menarche, late menopause, elderly pregnancy) can increase the risk of breast cancer [7,16,17].

Several other studies have shown that inflammation also correlates with cancer development. One research group combined inflammatory biomarkers, such as C-reactive protein (CRP) and leukocyte counts, to estimate the risk of cancer development. They found that inflammatory biomarkers were associated with cancer risk and mortality [18]. Another study showed that in addition to high-sensitivity C-reactive protein (hs-CRP) being able to evaluate the risk of cardiovascular diseases, increasing concentrations of hs-CRP in serum also correlated with prostate cancer risk and prognosis [19]. Abnormal levels of inflammation-related serum proteins, such as C-X3-C motif chemokine ligand 1, platelet-derived growth factor subunit B homodimer, interleukin 10, C-C motif chemokine ligand (CCL) 21, and CCL 11, were also found to be related to the risk of prostate cancer [20]. Taken together, these results suggest that inflammation increases the risk of various types of cancer.

Conversely, anti-inflammatory therapy might decrease the risk of cancer. Several cohort studies have shown that patients who received anti-inflammatory therapy had a decreased risk of developing certain cancers [8,9,10,21]. The risk of breast cancer was also shown to decrease for women who regularly used aspirin or other NSAIDs. All of the women had a history of breast cancer, with a large portion of them being *BRCA1* or *BRCA2* mutation carriers [9]. An early major prospective study of postmenopausal women in the Women’s Health Initiative (WHI) found clear evidence of chemoprevention of breast cancer by regular use of NSAIDs. By contrast, another study demonstrated that low-dose aspirin was not associated with reduced risk of breast cancer [12]. In addition, patients that regularly use NSAIDs may reduce their risk of certain subtypes (i.e., those with no BRAF/KRAS gene mutation) of colorectal cancer (CRC) [8]. The use of NSAIDs was also shown to decrease the risk of CRC in patients older than 40 years of age [11]. Moreover, the relationship of inflammation and breast cancer has been mentioned in several studies. The most popular enzymes found to be correlated with inflammation are cyclooxygenases (COXs). In the past two decades, two cyclooxygenases, that is, COX-1 and COX-2, have been observed to catalyze the conversion of arachidonic acid to prostaglandin endoperoxide (prostaglandin H2) [22]. This enzyme is responsible for the synthesis of various types of prostaglandin, which is an inflammatory factor [23]. Several studies have indicated that overexpression of COX-2, and to a lesser extent of COX-1, is directly associated with breast cancer [24,25,26]. Similar to COXs, lipoxygenase (LOX), which produces prostaglandins via a different pathway, has been found to be associated with breast cancer [27,28,29]. Taken together, overexpression of COXs and LOXs has been directly associated with breast cancer, which supports our results that mastitis increases the risk of breast cancer.

Our findings also suggest that hyperlipidemia could be another risk factor for breast cancer. One longitudinal analysis showed that hyperlipidemia was significantly higher in breast cancer patients with lymph node metastasis. The severity of hyperlipidemia was found to differ in proportion to tumor size and grade [30]. In addition, obesity has also been associated with various types of cancers, including breast cancer [31]. Conversely, cholesterol-lowering medication and physical exercise might lower the recurrence of breast cancer [32,33]. Hyperlipidemia may have a causal relationship with breast cancer.

Some studies have indicated that pregnancy could lower the risk of breast cancer. One possible reason for this is the maturation and differentiation of the mammary gland cells and breast tissue during the gestation period [34,35]. To eliminate pregnancy as a confounder, we included women whose age was ≥40 and excluded pregnant cases from one year prior to the study start date in both the mastitis and non-mastitis groups.

This study has several strengths. First, the LHID 2000 is a large dataset with representative data of the entire population of Taiwan. The utilization of the dataset should have prevented selection bias and enabled the identification of breast cancer and mastitis diagnoses from all sources. Second, the use of claims data prevented potential recall bias compared with self-reported history. Finally, methods used to avoid pregnancy would also affect the risk of breast cancer; therefore, in this study, we only included women whose age was ≥40 years and excluded women who had been pregnant in the year before observation. However, there are some limitations to our current study. First, the database used did not contain information regarding clinical parameters, such as abortion and menarche. In addition, NSAID use was not measured in the dataset. These factors may influence the risk of breast cancer. Second, we did not have access to potentially relevant personal behavioral information, such as smoking habits, alcohol consumption, sexual behaviors, and body mass index. These confounding factors might have affected the results of breast cancer risk. Third, Taiwan’s National Health Insurance system covers the population in Taiwan, and thus, our data accurately reflect Taiwan’s situation. However, our results may not be applicable to Western populations. Fourth, although the diagnosis of nonlactating mastitis is accurate, the limited ICD-9-CM code cannot further distinguish between acute and chronic states of mastitis; therefore, it remains unclear how that distinction may influence the risk of breast cancer.

## 5. Conclusions

In summary, this study supported the hypothesis that women who have had mastitis have a higher risk of developing breast cancer in the future. Our results also supported Nolan et al.’s hypothesis that mastitis is a risk factor for breast cancer [15]. Age and hyperlipidemia are other risk factors for developing breast cancer. Therefore, women aged ≥40 should take precautions to prevent mammary gland infections and to avoid obesity.

## Figures and Tables

**Table 1:** Demographic characteristics of mastitis and nonmastitis groups.

	Mastitis (N = 734)		Non-Mastitis (N = 7900)		
	*n*	%	*n*	%	*p*-Value
Age					0.272
40–50	390	53.1	4364	55.2	
≥50	344	46.9	3536	44.8	
Mean ± SD	51.1 ± 8.7		50.7 ± 8.8		0.195
Hypertension	151	20.6	1393	17.6	0.047
Hyperlipidemia	118	16.1	937	11.9	0.001
Diabetes	67	9.1	653	8.3	0.419
Hyperthyroidism	13	1.8	115	1.5	0.499
Hypothyroidism	10	1.4	73	0.9	0.244
Autoimmune diseases	18	2.5	183	2.3	0.815

**Table 2:** Cox proportional hazard model for risk of breast cancer.

	No. of Breast Cancer Events	Observed Person-Years	ID	CrudeHR	95% CI	*p* Value	aHR ^†^	95% CI	*p* Value
Mastitis									
No	36	20,576	1.7	1			1		
Yes	13	1918	6.8	3.87	2.05–7.3	<0.001	3.71	1.96–7.02	<0.001
Age									
40–50	26	12,250	2.1	1			1		
≥50	23	10,245	2.2	1.06	0.61–1.86	0.831	1.17	0.65–2.10	0.594
Hypertension	2	3979	0.5	0.20	0.05–0.82	0.025	0.14	0.03–0.58	0.007
Hyperlipidemia	10	2725	3.7	1.87	0.93–3.74	0.078	2.53	1.15–5.57	0.021
Diabetes	4	1871	2.1	0.99	0.35–2.74	0.980	0.89	0.29–2.74	0.835
Hypothyroidism	1	224	4.5	2.07	0.29–15.02	0.471	1.59	0.22–11.76	0.649
Autoimmune diseases	1	523	1.9	0.87	0.12–6.33	0.894	0.93	0.13–6.77	0.945

ID: incidence density (per 1000 person-years). HR: hazard ratio; aHR: adjusted hazard ratio. ^†^ Adjusted for age, hypertension, hyperlipidemia, diabetes, hypothyroidism, and autoimmune diseases.

**Table 3:** Subgroup analysis of risk of breast cancer.

	Mastitis		Non-Mastitis				
	N	No. of Breast Cancer Events	N	No. of Breast Cancer Events	HR	95% CI	*p* Value
Age							
20–50	390	5	4364	21	2.66	1.00–7.05	0.049
≥50	344	8	3536	15	5.46	2.31–12.87	<0.001
Hypertension							
No	583	12	6507	35	3.84	2.00–7.41	<0.001
Yes	151	1	1393	1	9.10	0.57–145.52	0.118
Hyperlipidemia							
No	616	9	6963	30	3.41	1.62–7.19	0.001
Yes	118	4	937	6	4.88	1.38–17.31	0.014
Diabetes							
No	667	10	7247	35	3.09	1.53–6.25	0.002
Yes	67	3	653	1	28.45	2.96–273.49	0.004
Hyperthyroidism							
No	721	13	7785	36	3.88	2.06–7.31	<0.001
Yes	13	0	115	0	NA	NA	NA
Hypothyroidism							
No	724	13	7827	35	4.00	2.12–7.56	<0.001
Yes	10	0	73	1	NA	NA	NA
Autoimmune diseases							
No	716	13	7717	35	4.00	2.12–7.56	<0.001
Yes	18	0	183	1	NA	NA	NA

NA: not applicable.
